# Supplementary material for: Predicting visual function by interpreting a neuronal wiring diagram
Source: Nature. 2024 Oct 2;634(8032):113–23. doi: 10.1038/s41586-024-07953-5 (PMC11446822; doi:10.1038/s41586-024-07953-5)
Supplement: Supplementary file 6 — CRF and ERF predictions for individual TmY4 and TmY9 cells. Analogous to Supplementary Data 3, but for TmY target types. Shown are the top four monosynaptic pathways, the strongest pathway passing through each of the top ten intermediary types (ranking from Extended Data Fig. 7), and the trisynaptic pathway Tm1–TmY–Dm3–TmY (see the section entitled Prediction of spatial normalization). [file 41586_2024_7953_MOESM6_ESM.zip › DataS4/TmY9q/720575940633195565.pdf]

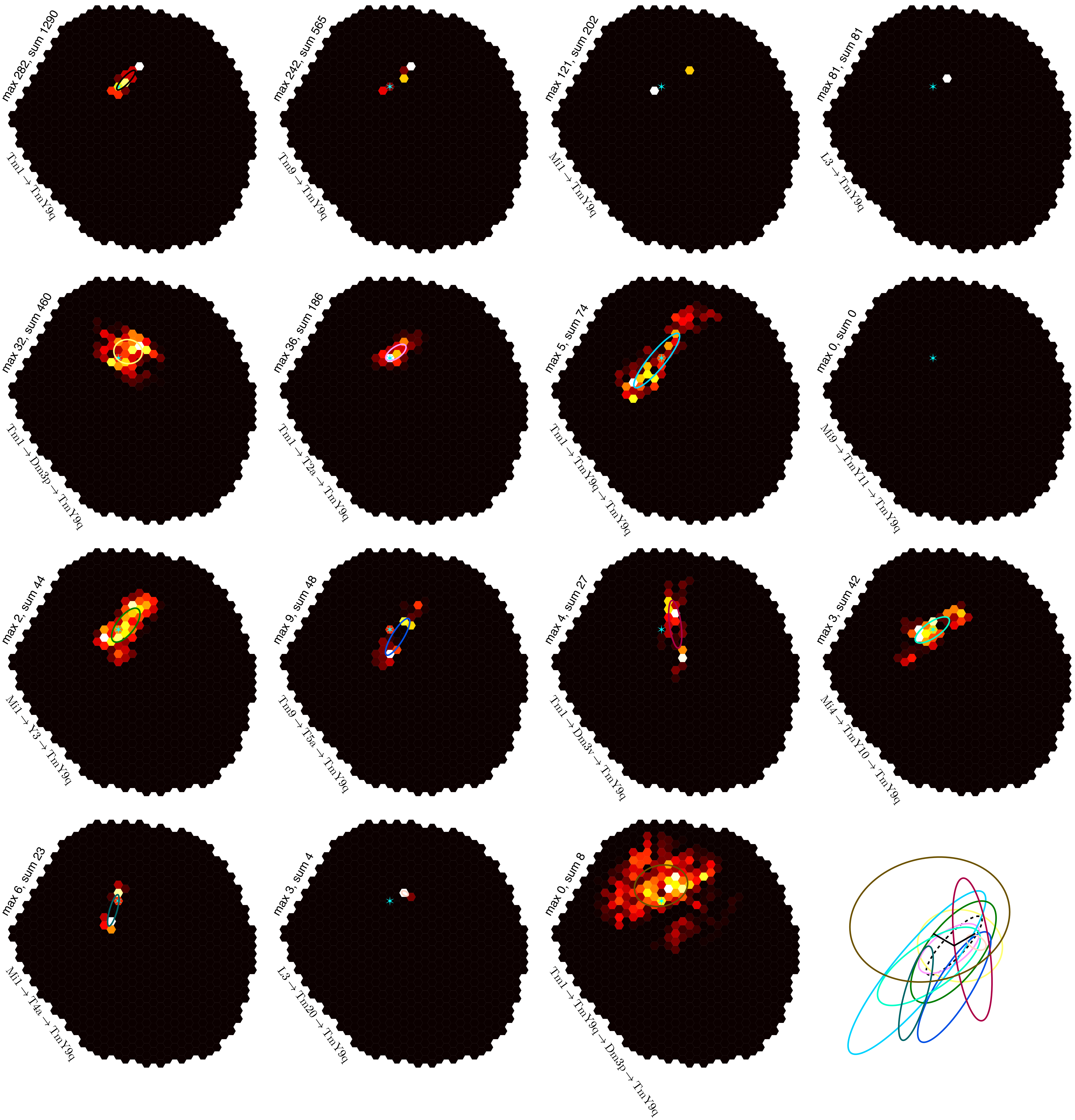

max 282, sum 1290
Tm1 → TmY9q
max 242, sum 565
Tm9 → TmY9q
max 121, sum 202
Mi1 → TmY9q
max 81, sum 81
L3 → TmY9q
max 32, sum 460
Tm1 → Dm3p → TmY9q
max 36, sum 186
Tm1 → T2a → TmY9q
max 5, sum 74
Tm1 → TmY9q → TmY9q
max 0, sum 0
Mi9 → TmY11 → TmY9q
max 2, sum 44
Mi1 → Y3 → TmY9q
max 9, sum 48
Tm9 → T5a → TmY9q
max 4, sum 27
Tm1 → Dm3v → TmY9q
max 3, sum 42
Mi4 → TmY10 → TmY9q
max 6, sum 23
Mi1 → T4a → TmY9q
max 3, sum 4
L3 → Tm20 → TmY9q
max 0, sum 8
Tm1 → TmY9q → Dm3p → TmY9q
